# The disappearing periglacial ecosystem atop Mt. Kilimanjaro supports both cosmopolitan and endemic microbial communities

**DOI:** 10.1038/s41598-019-46521-0

**Published:** 2019-07-23

**Authors:** Lara Vimercati, John L. Darcy, Steve K. Schmidt

**Affiliations:** 1 Department of Ecology and Evolutionary Biology, University of Colorado, Boulder, CO USA; 2 Department of Biomedical Informatics and Personalized Medicine, University of Colorado Anschutz Medical Campus, Denver, CO USA

**Keywords:** Soil microbiology, Biodiversity

## Abstract

Microbial communities have not been studied using molecular approaches at high elevations on the African continent. Here we describe the diversity of microbial communities from ice and periglacial soils from near the summit of Mt. Kilimanjaro by using both Illumina and Sanger sequencing of 16S and 18S rRNA genes. Ice and periglacial soils contain unexpectedly diverse and rich assemblages of Bacteria and Eukarya indicating that there may be high rates of dispersal to the top of this tropical mountain and/or that the habitat is more conducive to microbial life than was previously thought. Most bacterial OTUs are cosmopolitan and an analysis of isolation by geographic distance patterns of the genus *Polaromonas* emphasized the importance of global Aeolian transport in the assembly of bacterial communities on Kilimanjaro. The eukaryotic communities were less diverse than the bacterial communities and showed more evidence of dispersal limitations and apparent endemism. Cercozoa dominated the 18S communities, including a high abundance of testate amoebae and a high diversity of endemic OTUs within the Vampyrellida. These results argue for more intense study of this unique high-elevation “island of the cryosphere” before the glaciers of Kilimanjaro disappear forever.

## Introduction

High-elevation microbial diversity has been studied in several of the highest mountain ranges on Earth^1–3^, however microbial communities have not been studied at high elevation in the African continent and in particular, on the climate sensitive glaciers and periglacial soils on the top of Mt. Kilimanjaro. Kilimanjaro, Africa’s highest mountain and the tallest free standing mountain on Earth, is located on the Kenya-Tanzania border, about 370 km south of the equator and approximately the same distance from the Indian ocean (3 04′S, 37 21′E). This massive stratovolcano (about 80 by 50 km) consists of 3 main peaks, of which Kibo (5893 m) is the highest and the only one still retaining glaciers. Its summit has collapsed to form a caldera, enclosing the Reusch crater, which is about 800 m across and still presents a continuous geothermal heat flux. In recent years Kilimanjaro and its dramatic ice loss have become an “icon” of climate change, attracting broad interest in its fate^4^. The three remaining ice fields on the plateau and slopes are both shrinking laterally and rapidly thinning^5^ leaving behind just the ragged fringe of an ice cap, which is believed to have once covered the entire summit of the mountain^6^. Of the ice cover recorded in 1912, 85% has disappeared, with a peak reduction rate registered from 1989 to 2007 of about 2.5% per year^5^. A drastic drop in atmospheric moisture starting at the end of the 19^th^ century and an increase of shortwave incoming radiation due to decreased cloudiness are currently believed to be the main drivers of glacier retreat (through sublimation) on Kilimanjaro, as well as other equatorial east African glaciers^7^. It is believed that under present conditions, rapid glacier shrinking at the top of Kilimanjaro will continue unabated and the entire summit of the mountain is expected to be devoid of ice for the first time in 11000 years by mid-century^4^.

As the glaciers on top of Kilimanjaro continue to recede, unique microbial communities and paleodiversity archives will be gradually compromised and lost. Glaciers are known to function as reservoirs of airborne microorganisms that can preserve information about their relationship with climatic and environmental changes through time^8^. To date there have been no investigations published on the microbiology of the habitats at the top of Kilimanjaro and the expected dramatic change of these environments in the next decades makes it important to study the biological communities dwelling within the ice and soil. Its high elevation and its considerable isolation from any other mountain range also makes it an ideal site to further advance our knowledge of microbial endemicity and biogeographic patterns. Aerial deposition and post-depositional selection are the main drivers of microbial community composition at high elevations^8^ but relatively little is known about their specific roles in the establishment of microbial communities.

The top of Mt. Kilimanjaro is also of interest to the field of astrobiology. Kilimanjaro’s periglacial soils may be some of the most extreme environments on Earth and are therefore considered as potential analogues for habitable zones on Mars^9^. Microbial life at the top of this mountain has to cope with a complex interplay of parameters that are similar to those that could be found on Mars or other planetary bodies, such as a high UV flux, extreme diurnal freeze-thaw cycles, low atmospheric pressure and an extremely low nutrient content and water activity^9^. For this reason, these oligotrophic soils and glaciers have received some attention as Martian analogues prior to the launch of the Mars Science Laboratory (MSL) Curiosity Rover and as a field test for a planned Mars 2018 mission^9^. Preliminary data from Ponce *et al*.^9^ showed that Kilimanjaro soils in proximity to glacier walls may be nearly sterile with a total organic carbon (TOC) content of less than 1000 µg/g. TOC has been traditionally correlated with viable microbial biomass and the value observed for Kilimanjaro soils are slightly higher than TOC levels of oligotrophic mineral soils found in extreme environments in Antarctica, high elevation sites in the Andes and the Atacama Desert^1,10,11^. In the present study we follow up on the preliminary work of Ponce *et al*.^9^ to provide the first culture-independent analysis of the microbial communities in ice and soil on top of Mt. Kilimanjaro.

## Materials and Methods

### Site location and sample collection

**Figure 1:**
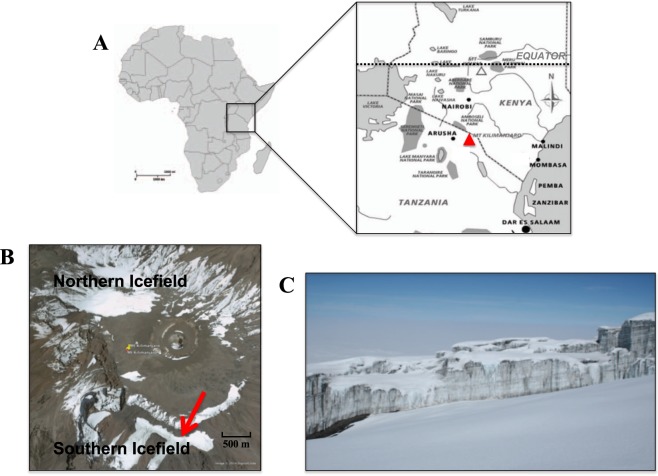
Location of Kilimanjaro sampling sites. (**A**) A map of Eastern Africa showing the location of Mt. Kilimanjaro on the Tanzania-Kenya border (red triangle). Mt. Kilimanjaro is located 370 km south of the Equator. (**B**) Google Earth imagery captured after field collections were made (Map data: Google, DigitalGlobe). Red arrow indicates sampling location at the border of the tabular-shaped plateau glacier in the Southern Ice Field at 5772 m elevation (S 03 04. 839′ E 037 21. 628′). (**C**) This panel features the tabular-shaped plateau glaciers of the Southern Icefield facing the sampling location and shows the large amount of snow cover that was present on the sampling day.

The study area at the top of Mt. Kilimanjaro was accessed from the Machame trail on June 4^th^, 2012, at the beginning of the dry season. Prevailing weather conditions at the top were clear, windy and cold, with most of the surface covered in fresh snow. Samples were collected at the border of the tabular-shaped plateau glacier in the Southern Ice Field (SIF) at 5772 m elevation (S 03 04. 839′ E 037 21. 628′) (Fig. 1). The collection site was far off the main trail with no apparent foot traffic. A total of 8 samples were collected along a transect from the base of the glacier wall: 2 from the ice wall (N3 and N8) and 6 from soils at increasing distance from the base of the glacier wall at distances of 0.4 m (N1), 2.5 m (N2), 5 m (N4), 7 m (N6), 7.5 m (N5) and 8 m (N8) (Table 1). Latitude, longitude and elevation data were collected using a handheld GPS device. The original plan to collect soil samples along a longer perpendicular transect was made impossible by deep snow on the ground, thus samples were collected from areas close to the glacier that were devoid of snow. Ice samples were collected by scraping the ice wall with a sterile spatula, while soil samples (50 g each) were collected from the top 0–4 cm of soil. Both types of sample were placed in sterile 50 ml centrifuge tubes (Fisher Brand, Hampton, NH, USA) and kept on ice for 1 day during the descent from the mountain. They were subsequently stored at −20 °C for a week and then shipped to the University of Colorado at Boulder. Upon arrival they were stored at −80 °C for later use in molecular and chemical analysis. A subset of collected samples has been archived at −80 °C for long-term storage to be available for potential future studies.

**Table 1:** Kilimanjaro sample sites and soil characteristics, sampled June 2012.

Sample	Location	Elevation	Distance from Glacier	Type	pH	DOC^a^ (µg/g soil)	TDN^b^ (µg/g soil^)^	% of H_2_O
N3	S 03° 04.839′ E 037° 21. 628	5772 m	on glacier wall	Ice				
N8	S 03° 04.837′ E 037° 21. 638′	5772 m	on glacier wall	Ice				
N1	S 03° 04.839′ E 037° 21. 628′	5771 m	0.4 m	Soil	7.5	44.8	25.3	28.1
N2	S 03° 04.839′ E 037° 21. 637′	5770 m	2.5 m	Soil	7.61	174.8	14.1	19.8
N4	S 03° 04.839′ E 037° 21. 636′	5772 m	5 m	Soil	7.49	22.3	9.5	13.6
N5	S 03° 04.839′ E 037° 21. 635′	5772 m	7.5 m	Soil	7.69	11.7	1.5	7.2
N6	S 03° 04.836′ E 037° 21. 634′	5772 m	7 m	Soil	7.93	7	3.1	7.7
N7	S 03° 04.835′ E 037° 21. 632′	5771 m	8 m	Soil	7.6	8.7	2.4	6.9

^a^DOC, Dissolved organic carbon.

^b^TDN, Total dissolved nitrogen.

### DNA extraction, PCR and 16S/18S rRNA gene clone library construction

Total environmental genomic DNA from soil and ice samples was extracted using PowerSoil^®^ and PowerWater^®^ DNA Isolation Kits respectively (MO BIO, Carlsbad, CA USA), according to the manufacturer’s instructions. PCR of 3 soil (N1, N5 and N7) and ice samples was performed using 1–3 µl of template DNA, 12.5 µl of AmpliTaq Gold® 360 MasterMix 5 Units/µL (Applied Biosystems, Foster City, CA, USA) and 1 µl of Forward and Reverse primers (10 µM) in a G-Storm GS2 thermal cycler (GRI Ltd., Essex, UK). Bacterial 16S rRNA genes were amplified using the bacterial domain-specific primer 8F (5′-AGA GTT TGA TCC TGG CTC AG-3′) and universal primer 1391 R (5′-GAC GGG CGG TGW GTR CA-3′). Eukaryotic 18S rRNA and archaeal 16S rRNA genes were amplified using the universal primer pair 4 Fa-short (5′-ATT CCG GTT GAT CCT GC-3′) and 1492r (5′-GGT TAC CTT GTT ACG ACT T-3′). PCR amplification for 16S rRNA genes was carried out using a program of 95 °C for 10 min followed by 35 cycles of 95 °C, 1 min; 53 °C, 30 sec; 72 °C, 2 min and 30 sec; with a final elongation step of 72 °C for 10 min. 18S eukaryotic and 16S archaeal rRNA genes were amplified by a program of 95 °C for 10 min followed by 35 cycles of 95 °C, 1 min; 49 °C, 30 sec; 72 °C, 2 min and 30 sec; and a final elongation step of 72 °C for 10 min. PCR products of the appropriate length were excised from agarose gels and purified following the protocol of the QIAquick Gel Extraction Kit (Qiagen, Valencia, CA USA), with Hyperladder II^TM^ as a reference. PCR products were ligated into TOPO TA® cloning vectors and transformed into OneShot^TM^ *E*. *coli* cells (Invitrogen, Carlsbad, CA, USA). Transformed cells were grown on Ampicillin 50 µg/mL agar plates overnight at 37 °C. Single colonies containing the inserts were pelleted and randomly arrayed on 96-well plates. Functional Biosciences (Madison, WI, USA) performed Sanger sequencing bi-directionally using vector-targeted T7 (5′-AAT ACG ACT CAC TAT AG-3′) and M13R-9 (5′-GCT ATG ACC ATG ATT ACG-3′) primers.

### Sanger sequencing and phylogenetic analysis

Geneious (Biomatters, Auckland, New Zealand) was used to edit sequences, trim primers and assemble contigs. Edited sequences were aligned using the SINA aligner tool^12^ and imported into ARB^13^ where they were manually fine-tuned. Putative chimeras were identified using a combination of Bellerophon^14^ and the Mallard Program^15^. Once putative chimeras were removed, remaining sequences were used to generate a phylip-formatted distance matrix and cluster analysis was performed with MOTHUR^16^ using the average neighbor algorithm implementation to define OTUs at the minimum threshold of 97% sequence identity. Phylogenetic affiliation of OTUs and related sequences were found using both the basic local alignment search tool (BLAST) and ARB through the parsimony insertion function. Phylogenetic trees for both bacterial and eukaryotic OTUs were inferred using maximum likelihood (ML). The ML analysis was conducted using a general time reversible (GTR) model of evolution with a gamma distribution (g) and a proportion of invariant sites (I) with MEGA6.06^17^. Node support was estimated using 300 bootstrap replicates.

### PCR and MiSeq Illumina sequencing

Amplification of the bacterial V4-V5 16S rRNA gene region and eukaryotic 18S rRNA gene for all soil and ice samples was performed using the oligonucleotide primers sets 515F/806R and Euk_1391f/EukBr respectively (Earth Microbiome Project, accessible at http://www.earthmicrobiome.org/emp-standard-protocols/16s-18s/). All forward and reverse primers were modified to include a unique 12 nucleotide barcode. PCR reaction mixtures contained 0.5 μL of forward primer (10 µM), 0.5 μL of reverse primer (10 µM), 1 μL of template and 12.5 μL of MM Gotaq Hot start Colorless Master Mix (Promega Corporation, Madison, WI, USA). The reaction volume was adjusted to a total of 25 μL with ultrapure DNase/RNase free water. Thermal cycles for 16S rRNA gene amplification consisted of an initial denaturation of 94 °C for 3 min, followed by 35 cycles of 94 °C for 1 min; 50 °C for 1 min; and 72 °C for 105 sec; with a final elongation step of 72 °C for 10 min. The program for 18S rRNA gene amplification consisted of an initial denaturation of 94 °C for 3 min, followed by 35 cycles of 94 °C for 45 sec; 57 °C for 1 min; and 72 °C for 1.5 min; with a final elongation step of 72 °C for 10 min. To prepare amplicons for sequencing, amplicon purification and normalization was done with Invitrogen SequalPrep Normalization Kit (Invitrogen Inc., CA, USA). Amplicons were combined into a single pool and sequenced using the Illumina MiSeq platform (BioFrontiers Institute, Boulder, CO) using pair-end 2 × 150 bp chemistry.

Forward-oriented sequences were demultiplexed, quality filtered and processed using the QIIME pipeline^18^. Paired-end sequences did not work for eukaryotic reads and only the read corresponding to the 1391 F primer was used because it overlaps more with sequences in the NCBI and SILVA databases^19^. Singletons were excluded from further analysis and sequences with >97% SSU rRNA gene sequence identity were clustered into an OTU via UCLUST. Representative sequences for each OTU were chosen for classification and the Greengenes and Silva 104 reference database for 16S and 18S rRNA gene sequences respectively were employed to assign taxonomy identification to each OTU. Sequences were aligned with PyNAST^20^ and a phylogeny was built with the FastTree algorithm^21^. OTU tables were rarified to the level of the lowest number of sequences in the lowest populous sample and were used to assess alpha diversity and relative abundance of all taxa.

### Diversity measures and statistical analysis

Diversity within individual communities (alpha diversity) was evaluated by measures of phylogenetic dominance, evenness and richness with MOTHUR^16^. Dominance was estimated through the Berger and Parker index^22^, which calculates the ratio between the abundance of the most represented OTU and the total OTU abundance. The Shannon Index was used to estimate community diversity taking into account OTUs richness and evenness. Phylotype richness was determined through the S_chao1_ estimator and an estimate of how well sampling had covered true community diversity was obtained with the non-parametric estimator Good’s coverage.

Weighted UniFrac Analysis, which quantifies shared evolutionary history through shared branch length^23^ was used to construct beta diversity matrices for both the ice and soil datasets (both 16S rRNA and 18S rRNA genes) and a one-way Analysis of Similarity test (ANOSIM) from the vegan R package^24^ was used to test significance of phylogenetic difference in beta diversity between ice and soil samples. Rarefaction curves were plotted for both bacterial and eukaryotic sequences with a sequence similarity cut-off value of 3% to evaluate if sampling effort had revealed true phylogenetic diversity within the site. Additionally, Principal Coordinate Analysis (PCoA) ordination was constructed based on weighted Unifrac distance matrices in order to visualize differences among community compositions of soil and ice samples.

### Environmental classification of OTUs

Environmental classification of long-read bacterial OTUs containing 2 or more sequences was done following a modified version of the method of Herbold *et al*.^25^. Each OTU representative sequence was queried against the NCBI nucleotide database using BLAST. OTUs were classified as “non-endemic” if closest matches to OTUs were >97% identical, “endemic” if closest matching sequences were <97% identical (Supp. Table 1). Within the “non-endemic” group, reads were classified as “cryophilic” if all database entries that matched the representative unique sequence with >97% identity had been previously observed in perennially cold environments, as “non-cryophilic” if all matches were observed in temperate environments and as “polythermal” if the matches were observed in both perennially cold and temperate environments. Reads for the endemic OTUs were classified as “subnovel” or “novel” if the nearest match was between 97% and 95.5% or 95.5–88.5% respectively^25^.

### Biogeographic analysis

In order to examine the biogeographic distribution within the cryosphere of the most abundant phylotypes retrieved on top of Mt. Kilimanjaro, a genetic isolation by geographic distance analysis was performed on the 2 most abundant long-read OTUs for which geographic coordinates of closely matching sequences were available in Genbank: a *Polaromonas* clade within the Bacteria (Supp. Fig. 1) and a *Chlamydomonas* clade within the Eukarya. Other long-read sequences from glacial and periglacial environments (Supp. Figs 2 and 3) closely matching the sequences of the clades were downloaded from GenBank (Supp. Tables 2 and 3). Geographic distances between sample sites were computed in R (R Development Core Team, 2012) using the Fields package (http://CRAN.R‐project.org/package=fields) and an uncorrected genetic distance matrix was created using the Ape package (http://CRAN.R‐project.org/package=ape). To test for a correlation between the matrices, Mantel tests were performed in R using 1000 randomized permutations per test. A Mantel correlogram was constructed and the application of Sturge’s rule resulted in data being partitioned in distance classes. Further Mantel tests were carried out on each distance class with a Bonferroni correction.

### Soil analyses

Soil pH was determined according to the method of King *et al*.^26^. Specifically, 2 g of soil and 2 mL of DI water were placed into 15 mL centrifuge tubes and shaken horizontally for 1 hr at 175 rpm. Soil pH was then measured with an Oakton benchtop pH meter (OAKTON Instruments, Vernon Hill, IL, USA). Total water content of soil samples was measured by placing 1 g of soil of each sample in 15 mL conical sterile tubes left open to dry at 60 °C in an oven for 24 hrs. Water content was determined as the percentage of the ratio between the water loss and dry weight of the sample.

Dissolved organic carbon (DOC) and total dissolved nitrogen (TDN) were measured using a modification of the method described in Weintraub *et al*.^27^. 2 g of soil of each sample was shaken with 25 mL of 0.5 M K_2_SO_4_ for 1 hour. Solutions were then filtered using a 0.2 µm Isopore^TM^ membrane filter (Millipore, Darmstadt, Germany). DOC and TDN were measured using a Shimadzu TOC-V CSN Total Organic Carbon Analyzer with TNM-1 module.

### Nucleotide sequence accession numbers

The SSU rRNA gene sequences from Sanger libraries from this study were deposited in the GenBank database under accession numbers KX771236 to KX772158 while the SSU rRNA gene sequences from Illumina MiSeq libraries were deposited in the SRA (Short Read Archive) database under Bioproject ID PRJNA340181 and PRJNA340027.

## Results

### Alpha and beta diversity

Bacterial richness (Chao 1) and Alpha Diversity (PD) were comparable for soil and ice samples (Table 2), whereas the eukaryotic community showed more diversity in ice compared to soils. The Shannon Index implied than evenness was similar in soils and ice for both bacterial and eukaryotic communities. The Berger Parker Index revealed relatively low dominance in both ice and soil bacterial communities, meaning that the most abundant OTUs in the samples are only represented by a small number of sequences. Eukaryotic communities in soil and ice displayed a higher Berger Parker Index than that seen for bacterial communities showing that the most abundant OTUs constitute 40–50% of total abundance.

**Table 2:** Diversity and dominance indices, coverage, number of observed OTUs and phylogenetic diversity index (PD) for bacterial and eukaryotic communities in soil and ice samples.

Sample	Chao1	Shannon	Berger-Parker	Good’s Coverage	Observed OTUs	PD
**Bacteria**						
Soil	603.37	6.37	0.12	0.97	418	20.74
Ice	646.83	6.32	0.09	0.97	400	19.89
**Eukarya**						
Soil	184.36	2.65	0.47	0.99	113.3	38.48
Ice	284.85	2.73	0.39	0.98	143.5	45.59

Rarefaction curves (Supp. Fig. 4) yielded similar patterns as those estimated by Chao1 index and were close to saturation at a 97% clustering level. The number of OTUs observed was ~3.5 times higher for Bacteria compared to Eukarya. Within the Eukarya, sampling coverage for ice communities was about twice as high as soil communities. Good’s coverage index demonstrated that the estimated proportion of phylotypes represented in our libraries was very high (>0.97), however, “species-level” sampling coverage was not complete as the observed number of OTUs was less than that estimated with the Chao1 richness.

ANOSIM analysis revealed that there was a significant difference in composition (beta diversity) between ice and the soil samples for Bacteria (R = 0.8, P = 0.03), but there was not a significant difference between ice and soil eukaryotic assemblages, perhaps indicating that the soil Eukarya came from the ice. Interestingly both the bacterial and eukaryotic composition of the soil sample closest to the ice wall (N1) was more similar to that of ice samples compared to soil (Supp. Fig. 5). Ice assemblages showed a lower weighted UniFrac dissimilarity value to the soil sample closest to the glacier wall than the other soil samples for both Bacteria (0.13 vs 0.202) and Eukarya (0.212 vs 1.49).

### Community composition and phylogenetic analysis

**Figure 2:**
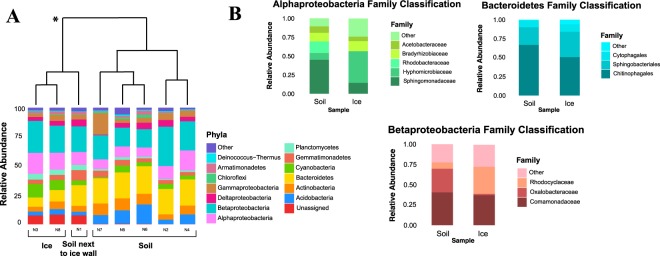
Phylogenetic analysis of 16S rRNA gene sequences obtained from ice and soil samples close to the summit of Mt. Kilimanjaro. (**A**) Cluster analysis of the phylogenetic structure and the relative abundance of bacterial phyla in the ice and soil samples as assigned by Greengenes to Illumina MiSeq sequences. Proteobacteria were split into classes for greater detail. The asterisk indicates statistical significant difference in composition (ANOSIM, P < 0.05). (**B**) Classification at lower taxonomic level is reported for Alphaproteobacteria, Bacteroidetes and Betaproteobacteria.

Bacterial OTUs from soil and ice were distributed among 18 phyla, including 4 candidate divisions and were dominated in both ice and soil by Proteobacteria, Bacteroidetes, Actinobacteria and Acidobacteria (Fig. 2). Betaproteobacteria represented the dominant fraction of the community in both ice (25%) and soil (22%) and most of these were in the Comamonadaceae (*Polaromonas*, *Variovorax*, *Rhodoferax*, *Curvibacter*, *Methylibium*) and Oxalobacteraceae in the soil and Rhodocyclales in the ice. Bacteroidetes accounted for 9 and 22% in ice and soil, respectively, with the majority in the Chitinophagaceae family (*Ferruginibacter*, *Segetibacter* and *Sediminibacterium*). Actinobacteria accounted for 5 and 8% of the total sequences in ice and soil, respectively, with most being in the Actinomycetales (*Frigobacterium*, *Nocardioides* and *Pseudonocardia*). Cyanobacteria only represented 4% of sequences in the soil, whereas they were more abundant in the ice (10%), with most sequences in the Oscillatoriales and Chroococcales. Overall soil communities harbored higher abundances of Actinobacteria, Bacteroidetes and Acidobacteria than ice, while sequences related to Betaproteobacteria, Alphaproteobacteria and Cyanobacteria displayed higher abundance within ice communities (Fig. 2).

**Figure 3:**
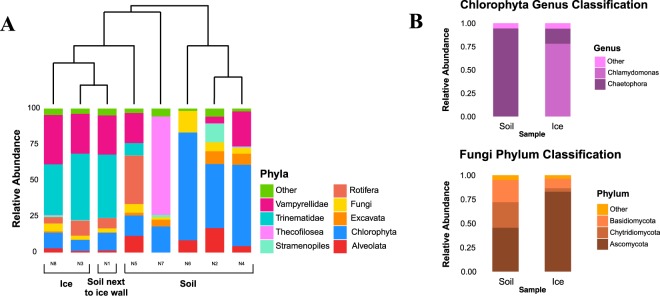
Phylogenetic analysis of 18S rRNA gene sequences obtained from ice and soil samples close to the summit of Mt. Kilimanjaro. (**A**) Cluster analysis of the phylogenetic structure and the relative abundance of eukaryotic phyla in the ice and soil samples as assigned by Silva 104 database to Illumina MiSeq sequences. Cercozoa were split into classes (Thecofilosea), orders (Vampyrellida) and families (Trinematidae) for greater detail (**B**) Classification at lower taxonomic level is reported for Chlorophyta and Fungi.

Illumina sequencing data revealed that both soil and ice 18S communities were limited to 9 phyla. Ice communities were heavily dominated by Cercozoa, representing 72% of total sequences (Fig. 3), while in the soil they were the most abundant taxonomic group together with green algae (Chlorophyta), at about 34% abundance each. The Chlorophyceae comprised about 9% and 34% of total sequences in the ice and soil respectively and were most closely related to the genera *Chlamydomonas*, *Chloromonas* and *Stigeoclonium*.

**Figure 4:**
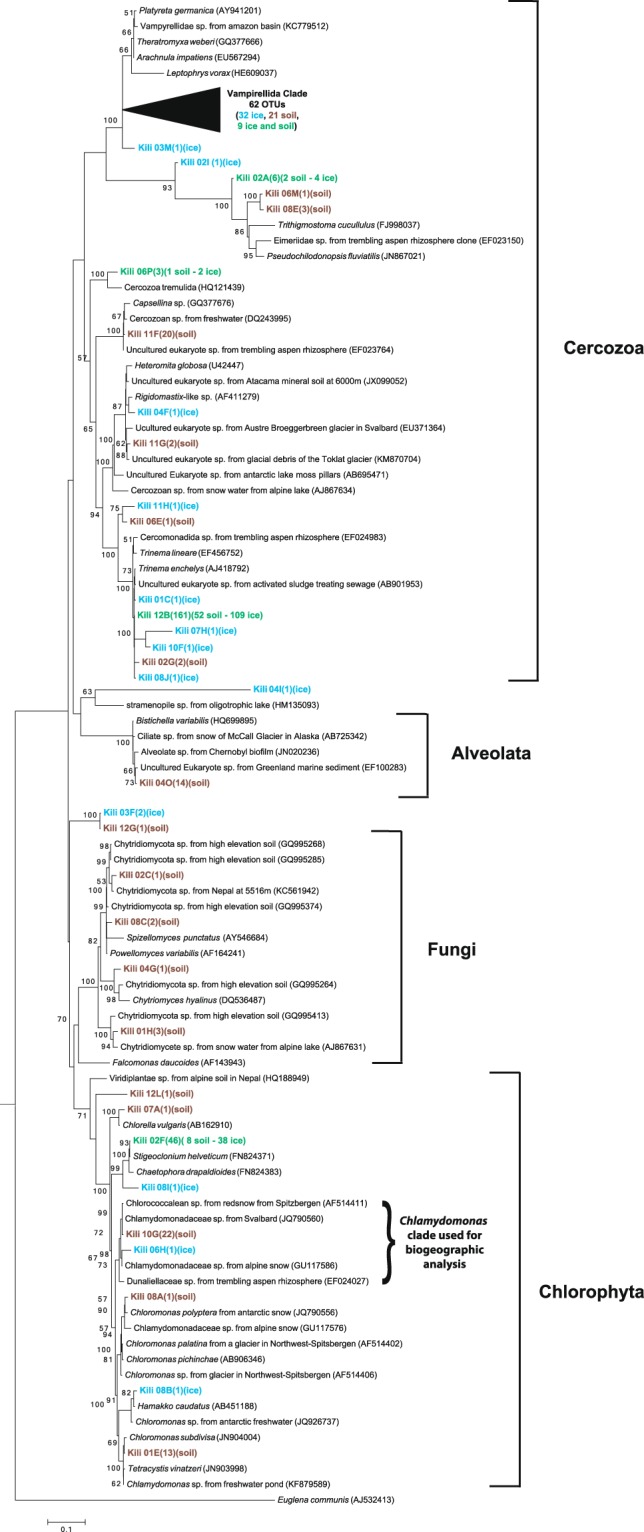
Phylogenetic analysis of 18S rRNA gene long-read sequences retrieved in ice and soil samples close to Mt. Kilimanjaro summit. Maximum Likelihood consensus phylogenetic tree includes 18S rRNA gene sequences from ice and soil close to the summit of Mt. Kilimanjaro and their closest GenBank BLAST and ARB matches. The accession numbers of most closely related taxa are listed parenthetically. Tree is rooted with the sequence of *Euglena communis* (AJ532413). Kilimanjaro phylotypes are bolded and followed by the number of sequences in each phylotype. Kilimanjaro phylotypes color code is as follows: blue, sequences retrieved in ice: brown, sequences retrieved in soil: green, sequences retrieved in both ice and soil. Node support is given as maximum likelihood values (n. of bootstrap replicates) when equal or greater than 50%. The scale bar corresponds to 0.1 substitutions per site. Major groups are shown to the right. The parenthesis indicates the *Chlamydomonas* clade that was used for the biogeographic analysis.

Within the Cercozoa, most sequences fell within the Trinematidae and the Vampirellida. Longer reads from Sanger sequencing showed that the Trinematidae family clustered in very few OTUs closely related to the known organisms *Trinema lineare* and *Trinema enchelis* (Fig. 4). In contrast, long-read sequences within the Vampirellida order were very diverse and all fell into a previously undescribed clade (wedge in Fig. 4).

A total of 15 archaeal Sanger sequences that clustered into 3 OTUs were also retrieved in one of the soil samples (N7) with closest relatives being uncultured Haloarchaea from alkaline-saline desert and Antarctic soils (Supp. Fig. 6), however we did not use Archaea-specific primers in this study.

### Environmental classification of OTUs

We used the approach of Herbold *et al*.^25^ to classify bacterial phylotypes into “endemic” and “non-endemic” and within the non-endemic whether they are “cryophilic”, “non-cryophilic” or “polythermal” (Supp. Table 1). Of the 60 non-endemic bacterial OTUs found on Mt. Kilimanjaro summit, 46 (77%) were found to be cosmopolitan, meaning that all database entries that matched the representative sequence for that OTU had been previously observed in both perennially cold and temperate environments. Only 6 bacterial OTUs appeared to be endemic, meaning that they were less than 97% identical to any phylotype in the NCBI database.

### Biogeographic analysis

**Figure 5:**
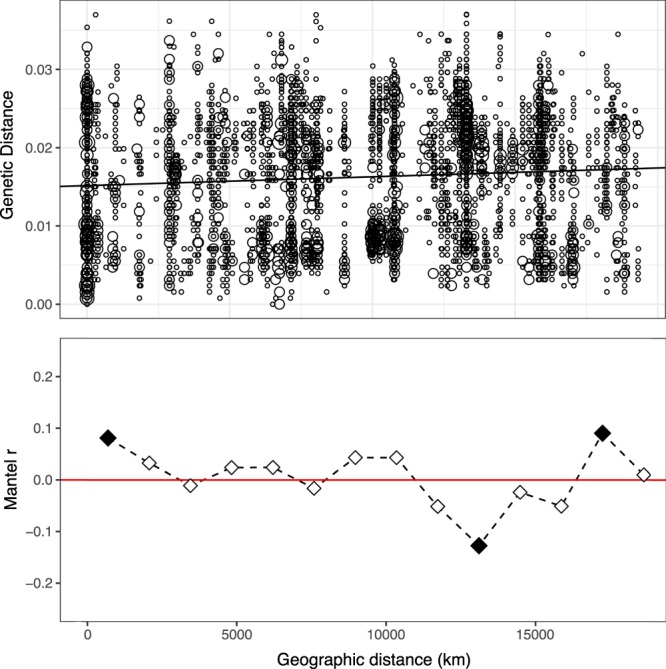
Genetic distance by geographical distance relationships for the dominant *Polaromonas* clade from Mt. Kilimanjaro summit. Upper panel shows pairwise comparisons of genetic distance among *Polaromonas* sequences (n = 4560) and geographic distance among different glacial environments (n = 25). The largest geographical distance in this study was between John Evans Glacier, Nunavut and Kamb Ice Stream, Antarctica (19307 km). There was a slight (rM = 0.08) but significant (Mantel test, P = 0.002) increase in genetic distance with geographic distance. Circle size is proportional to the number of pairwise comparisons at each point on the plot. Lower panel shows Mantel correlogram of genetic distance among the same sequences separated into 14 distance classes. The midpoint of each distance class is plotted. Shaded squares indicate significant tests in the Mantel correlogram after applying the Bonferroni correction. Spatial structuring was not evident for 11 out of 14 distance classes, supporting the contention that *Polaromonas* phylotypes are globally distributed.

**Figure 6:**
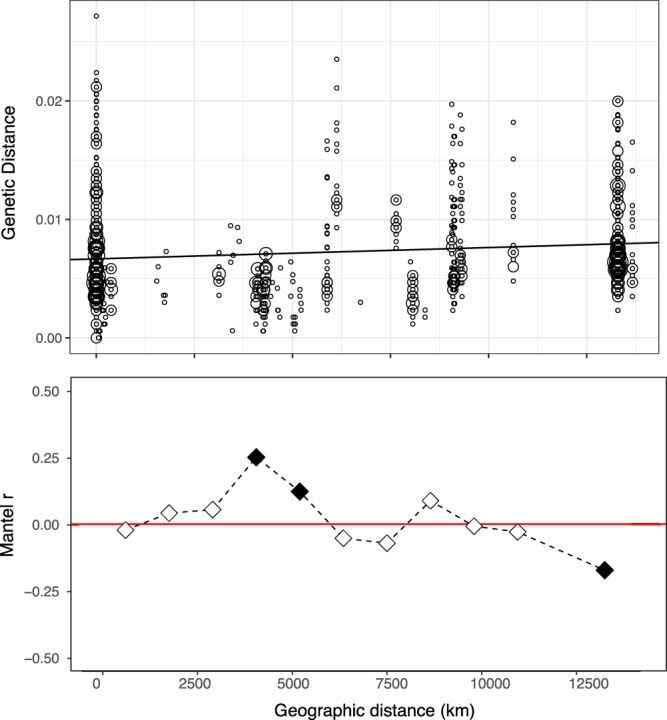
Genetic distance by geographical distance relationships for the dominant *Chlamydomonas* clade from Mt. Kilimanjaro summit. Upper panel shows pairwise comparisons of genetic distance among *Chlamydomonas* sequences (n = 1326) and geographic distance among different glacial environments (n = 15). The largest geographical distance in this study was between Kilimanjaro and Harding icefield, Alaska (13667 km). There was a slight (rM = 0.125) but significant (Mantel test, P = 0.001) increase in genetic distance with geographic distance. Circle size is proportional to the number of pairwise comparisons at each point on the plot. Lower panel shows Mantel correlogram of genetic distance among the same sequences separated into 11 distance classes. The midpoint of each distance class is plotted. Shaded squares indicate significant tests in the Mantel correlogram after applying the Bonferroni correction. Spatial structuring was not evident for 8 out of 11 distance classes.

Within the Bacteria and the Eukarya, two genera common to glacial and periglacial environments were selected to test their biogeographical distribution by comparison of genetic distance versus geographic distance. The analysis of the *Polaromonas* clade revealed that there was a slight (r_M_ = 0.08) but significant (Mantel test, P = 0.002) increase in genetic distance with geographic distance, however spatial structuring was not evident for 11 out of 14 distance classes (Fig. 5). An expanded dataset of Darcy *et al*. 2011 was used (Supp. Table 2) including 96 long-read sequences from 25 geographically well separated glacial and periglacial environments (Supp. Fig. 2), which makes it the most inclusive long-read *Polaromonas* biogeographical analysis performed to date. The biogeographic analysis on the *Chlamydomonas* clade showed a slightly stronger (r_M_ = 0.125, P = 0.001) increase in genetic distance with geographic distance, but spatial structuring was not evident for 8 out of 11 distance classes (Fig. 6).

### Soil characteristics

Soil moisture levels decreased with distance from the glacier wall (Table 1) and are comparable with other high elevation and periglacial sites^1,28^. Soil pH did not show a trend and ranged from 7.5 to 7.9 across the transect (Table 1). DOC and TDN away from the glacier (>5 m) are similar to levels found for Antarctic bare, fell-field soils^29^ and high elevation sites in Peru and Colorado^30^. In general, nutrient levels were higher in samples taken close to the glacier, especially TDN (Table 1).

## Discussion

Despite their ecological importance to downstream ecosystems, the study of microbial diversity at high elevations has lagged behind the study of other ecosystems. In particular, no work, using modern molecular approaches, has been done at high elevations in Africa. The present study was initiated to probe the diversity of soil- and ice-dwelling microbes near the top of Mt. Kilimanjaro. To our knowledge, this is the first investigation of the microbial diversity on Mt. Kilimanjaro or the African continent at elevations above 5000 m. This investigation provides a reference for future studies of microbial community structure and function in this extreme environment and other high-elevation sites in Africa.

Data from Illumina and Sanger libraries demonstrated that communities in both soil and ice are represented by a phylogenetically broad spectrum of microorganisms. Richness was high for both soil and ice communities for Bacteria (Table 2) and was close to that previously reported for other cold environments such as Antarctica and the Himalayas^31,32^. Glaciers can support very active microbial communities that sequester nutrients from the atmosphere^33–35^, which may explain the high diversity encountered in ice samples. The majority of bacterial OTUs were not closely related to cultured microorganisms and among the closest matching sequences were environmental sequences from cold (high-elevation and high-latitude), arid and heavy metal contaminated environments. These comprised putative psychrophilic, xerophilic, endolithic, radioresistant and halophilic microorganisms (Supp. Fig. 1). Diversity of Eukarya was a third of that for Bacteria according to the Shannon Index and Observed OTUs (Table 2), but was comparable to Eukaryotic diversity in polar waters^36^ and sea ice^37^.

Bacterial communities in ice and soil samples were significantly different (ANOSIM, P < 0.05). However, the soil sample closest to the ice wall harbored bacterial and eukaryotic communities closely resembling those of the ice samples. This may suggest that the main microbial input for the soils adjacent to the glaciers is the glacier itself and that these ice microbes are replaced by a soil community as the soils age. Ice microorganisms may not survive as well in soils because of exposure to environmental stressors such as freeze-thaw cycles and desiccation, stressors that are partially alleviated in the more constant ice environment. A likely consequence may therefore be the establishment in the soil of new phylotypes from the atmosphere.

### Taxonomic diversity of bacteria

Overall, both Illumina and Sanger technologies yielded similar patterns of taxonomic diversity (Supp. Fig. 7) as has been noted in other studies^38^ with the taxonomic resolution achieved dependent on the abundance of reference sequences in the particular region of the rRNA tree of life. Sanger sequences were much longer (1345–1715 vs 150–250 bp) and were therefore used to identify sequences at a finer taxonomic level.

Betaproteobacteria (especially members of the Comamonadaceae) are the dominant members of the community similar to other recently deglaciated soils^39–41^. Comamonadaceae are phylogenetically similar in soil and ice (Supp. Fig. 1), indicating that ice may be seeding the youngest soils as previously seen in the High Andes of Peru^40^. Many Comamonadaceae are heterotrophs that are able to metabolize both recalcitrant^42^ and more labile carbon sources^43^ and could therefore use multiple aeolian deposited carbon sources. It is still unclear if many Comamonadaceae (e.g. *Polaromonas*) are indigenous to ice or if they are transients from the upper atmosphere. They are found in almost all glacial habitats studied to date^39^, perhaps indicating that they are being constantly deposited in glacial systems, but not necessarily growing there.

The other most abundant bacterial OTUs in soil were in the Chitinophagaceae (Bacteroidetes) but these were in lower relative abundance in the ice. Closely related sequences have been previously retrieved from similar environments such as oligotrophic volcanic cave sediments from Mt. Erebus, Antarctica^44^, fumarole soils at high elevation in the Andes^1^, pyroclastic deposits in Alaska^45^, and debris-covered glaciers in the Alps^46^. Chitinophagaceae have the ability to degrade complex polymers such as chitin, which provides microbes with C and N^47^. Chitin may come from Fungi and Arthropods, which often occur in recently deglaciated soils^48^. Members of the Chitinophagaceae may be opportunists, relying on Aeolian inputs and some species have a dormant stage^49^, which may allow them to persist in the atmosphere and survive in periglacial habitats once they are deposited.

All cyanobacteria sequences retrieved in ice and soil are closely related to non-heterocystous nitrogen fixers (*Oscillatoria*, *Microcoleus*, *Leptolyngbya*) commonly found in cold ecosystems^50^. Nitrogen is often a limiting nutrient in poorly developed early successional soils^51^. Other sites at similar elevations in Peru and Nepal have also recently been shown to have similar cyanobacteria^3,40,52^ and cyanobacteria have long been known to colonize barren habitats in the Arctic and Antarctica^53^. However, very dry, high-elevation sites like the stratovolcanoes in the Atacama Desert completely lack cyanobacteria, except in wet areas near active fumaroles^1,2,54^. Cyanobacteria were more abundant in the ice wall and the soil adjacent to the glacier than soil, possibly indicating that they depend on access to a constant source of water (provided by meltwater of the glacier), which is not available in soils further from the glacier.

Among the Alphaproteobacteria, the most abundant Hyphomicrobiaceae family was only found within ice samples and the soil closest to the ice wall and are known for being oligocarbophilic, thriving only in the presence of low carbon concentrations^55^, which can explain their being restricted to the ice and soils near the ice. Sphingomonadaceae were the second most abundant family within the Alphaproteobacteria and were more abundant in soils. Sequences from this family are commonly detected in glacial habitats and have been reported in Arctic cryoconite^56^, Antarctic ice sheet^57^ and Arctic glaciers^58^. Members of the Deinococcus-Thermus group were present in our site, as well as Antarctica Dry Valleys^59^ and sites over 5400 meters in the Andes^60^.

### Taxonomic diversity of eukarya

Among the Eukarya, Cercozoa dominated the community and the most unexpected diversity found was in the order Vampyrellida (family Leptophryidae). These sequences are distinctly different from any taxa in Genbank (Fig. 4), suggesting that this unique environment harbors a previously unknown Vampyrellida community. Vampyrellida are only rarely recovered in environmental DNA surveys^61^ and have only been previously found at high elevations in soils from fumaroles on Volcán Socompa^62^. Our sequences were however less than 95% identical to those found on Volcán Socompa suggesting high endemicity of this group on Kilimanjaro. Given the presence of fumarolic activity on Mt. Kilimanjaro, it is possible that this group is associated with thermal environments. The Vampyrellida are very diverse and are generally known from aquatic habitats and soil where they are predators of algae, fungi, protozoa and small metazoans^63^. Vampyrellida have multiple nuclei^63^, which could lead to a bias and overestimation in their relative abundance. However, our Sanger clone libraries showed many distinct OTUs suggesting that multiple representation of the same organism was minimal. Many Cercozoa (including some Vampyrellida) are very resistant to drought^64^ and can respond quickly to periods of transient nutrient and water availability^63^, which may account for their abundance in the soils studied here. Food preferences may also be an important basis for lineage differentiation in Vampyrellida^65^. Their relative abundance was significantly higher in the ice samples (Fig. 3 and Supp. Fig. 8), suggesting that those in the soil may have come from the ice.

The most abundant OTU in the Eukarya was very closely affiliated with the known Cercozoans *Trinema lineare* and *Trinema enchelis* (order Euglyphida)^66^, common testatae amebae in moist environments. The same taxa of testatae amebae have recently been found for the first time in glaciers in the Andes^67^ and in glacier forefield soils in the Alps^68^. Heterotrophic Cercozoa seem to be more abundant than their potential food sources in our samples. However, inverted trophic pyramids have been seen before at high elevation where producers are scarce^1^ or absent^2^. The high abundance of these predatory microeukaryotes could be sustained by feeding on fungi and algae in addition to bactivory^69^.

These soils, like other even higher-elevation volcanoes^1,2^, harbored some of the simplest fungal communities yet described compared to, for example the 15 lineages recovered in the Dry Valleys of Antarctica^70^. Environmental extremes characteristic of high elevation, such as freeze-thaw cycles and high UV levels may limit fungal communities on Kilimanjaro compared to polar regions. Chytrids dominated our Sanger fungal libraries similar to previous studies of high-elevation and high-latitude soils^71^. Most chytrids were in the Spizellomycetes, which are commonly found in harsh soil environments such as arid grasslands^72^, and glacial till^73^. They can resist extremes of temperature, pH, and desiccation^74^ and can decompose pollen or be parasitic. These features may explain their presence on Mt. Kilimanjaro and their wide geographical distribution.

Green algae sequences found in ice and soils of Kilimanjaro are closely related to those found in similar environments such as alpine snow^75^, Arctic glaciers^76^ and Antarctic snow^77^. Their high relative abundance and the scarcity of cyanobacterial sequences in these soils suggests that they may play a significant role as primary producers in this habitat and in early stages of succession following glacial retreat as has been previously suggested for green algae in high-altitude ecosystems^78^.

### Taxonomic diversity of archaea

Archaea sequences distantly related (92%) to the Haloarchaea class were only retrieved with Sanger sequencing in one of the soil samples (N7) (Supp. Fig. 6). Scarcity of liquid water at this elevation can drive the formation of salty pockets within the soils that can explain the presence of Haloarchaea but more work is needed to understand the true phylogenetic diversity of Archaea on the top of Mt. Kilimanjaro. It is known that Archaea are more difficult to detect in cold^79,80^ and hot deserts^81^, which may explain our findings.

### Biogeography and spatial scale

Kilimanjaro is the most isolated high-altitude peak on Earth^82^ and approximately 5700 km from the closest mountain range with similar and higher elevation peaks (the Himalayas) (Google earth data, last accessed on 10 March 2018) making this site ideal for testing ideas related to microbial dispersal and biogeography. Despite being separated by large distances and climatic barriers, it has been proposed that cold high-elevation and high-latitude environments are connected through the upper atmosphere^39^ but data is lacking for Africa. Small microbes are easily dispersed over long distances, but their long-term survival requires a number of distinct adaptations, similar to those required to survive in icy environments^83^. The top of Mt. Kilimanjaro may be an oasis where microbes in transit in the upper atmosphere persist, but more work is needed to determine if they can grow on Kilimanjaro. The present study is a first step towards gaining an understanding of the global dispersal of microbial phylotypes to high elevation sites in Africa.

It is currently unknown how Aeolian deposition affects microbial composition and if Kilimanjaro’s great distance from similar environments is reflected in more speciation. Our survey showed that most bacterial diversity at our site is cosmopolitan, as defined by Herbold *et al*.^25^, with the vast majority of OTUs having close matches with sequences from very distant both cold and temperate environments showing little endemism in this cold isolated environment. More evidence of minimal endemism of this isolated site comes from the biogeographical analysis of one of the most abundant clades within the Bacteria, *Polaromonas* (Fig. 5). Our analysis showed remarkably little increase in genetic diversity with geographic distance, pointing towards a uniform distribution of *Polaromonas* phylotypes across the cryosphere. These results confirm the conclusion of previous work that *Polaromonas* phylotypes are globally distributed in the cryosphere^39^. Interaction with local microbiota may drive microevolution and post-selection niche separation within *Polaromonas* on the top of Mt. Kilimanjaro.

The finding in Kilimanjaro samples of both cosmopolitan and endemic microorganisms helps corroborate the hypothesis that the distribution of microbial species in the cryosphere is the outcome of the continuum between two opposing theories in microbial ecology: endemism and cosmopolitanism^84^. Moreover, the closest matches of 14% of sequences retrieved from our samples were from heavy metal contaminated sites. Since our site is not known to contain relevant quantities of heavy metal, it may be possible that tolerance and resistance mechanisms to heavy metals overlap with those employed by microorganisms to cope with stresses found in extreme environments such as high radiation, freeze-thaw cycles, low nutrient and water levels^81,85^.

It is likely that larger eukaryotic microbes might be more dispersal limited than smaller Bacteria. Previous work in the cryosphere has shown that larger microbes like algae and zoosporic fungi show distinct biogeographic patterns at global and regional scales^3,86,87^ and this could be the case for the most abundant phylum retrieved from our samples, the Cercozoa, which showed a highly diverse new clade within the Vampyrellida. Successful dispersal of small microbes is due to their high abundance and long residence time in the atmosphere^88^, which may not be the case for eukaryotic microorganisms due to their average larger size. Evidence of a pattern of genetic isolation by genetic distance was revealed by the phylo-geographic analysis of a highly abundant *Chlamydomonas* clade. However, despite the large distances, the pattern revealed was weak (Mantel rM = 0.125, P = 0.001) and it is in accordance with recent molecular studies that suggest that red snow algae may be readily dispersed across the Earth^89,90^. Our analyses suggest that *Chlamydomonas* may be successful at global dispersal, however in a more limited fashion compared to most bacteria.

### Conclusions and implications

Microbial diversity in the extreme environments on top of Mt. Kilimanjaro proved to be higher than expected compared to high volcanoes in drier regions^1,2,54^, especially for the Bacteria. In addition, the ice bacterial community was significantly different from the soil community, suggesting that soils are predominately seeded by wind-transported terrestrial sources. The combination of cosmopolitan bacterial diversity and weak biogeographical patterns for the clades analyzed suggest that the effect of distance and local environmental conditions is overwhelmed by continuous dispersal. Taken together, considering the isolation of Mt. Kilimanjaro from other perennially cold environments, our results strongly support the concept of a global distribution of microbial ecotypes throughout the cold terrestrial biosphere as has been suggested by other studies of Bacteria^3,39,50^. This finding suggests that there may be gene flow between the polar regions or that the rate of evolutionary divergence has been slow relative to the timescales of isolation. In depth molecular comparisons of microbial diversity with other distant high elevation sites could provide further insights into dispersal mechanisms and may help elucidate how microbes adapt and diversify in response to environmental pressures. Unravelling the full extent of diversity in these environments would provide a unique opportunity to investigate microbial endemism and evolution as well as roles of inoculum from the atmosphere, fumaroles and glaciers for microbial communities in recently deglaciated soils.

As glacier recession continues unabated, understanding the biodiversity and function of organisms in these glaciers and periglacial soils is important, before these ecosystems are changed forever. One of the main concerns is the loss of biodiversity and potentially the loss from the biosphere of a pool of genes adapted to surviving and thriving in the cold^91^. Our work adds to a growing body of knowledge about microbial diversity in high elevation ecosystems that are currently responding to a rapidly changing climate. The presence of high bacterial and eukaryotic diversity indicates that specific life forms have evolved to thrive in this extreme environment and may provide a model for viable habitats for life on early Mars since similar conditions may have existed or still exist in sediments adjacent to and beneath the Martian north polar ice cap^9,92^.

## Supplementary information

Supplementary Info
