# Supplementary material for: The disappearing periglacial ecosystem atop Mt. Kilimanjaro supports both cosmopolitan and endemic microbial communities
Source: Sci Rep. 2019 Jul 23;9:10676. doi: 10.1038/s41598-019-46521-0 (PMC6650471; doi:10.1038/s41598-019-46521-0)
Supplement: Supplementary file 1 — Supplementary Info [file 41598_2019_46521_MOESM1_ESM.pdf]

The disappearing periglacial ecosystem atop Mt. Kilimanjaro supports both cosmopolitan and endemic microbial communities

Lara Vimercati[a],

John L. Darcy[b],

Steve K. Schmidt[a#] (corresponding author)

[#]Address correspondence to steve.schmidt@Colorado.edu

Department of Ecology and Evolutionary Biology, University of Colorado, Boulder, CO, USA[a]

Department of Ecology, Evolution and Conservation Biology, University of Hawai'i at Manoa, Honolulu, HI, USA[b]

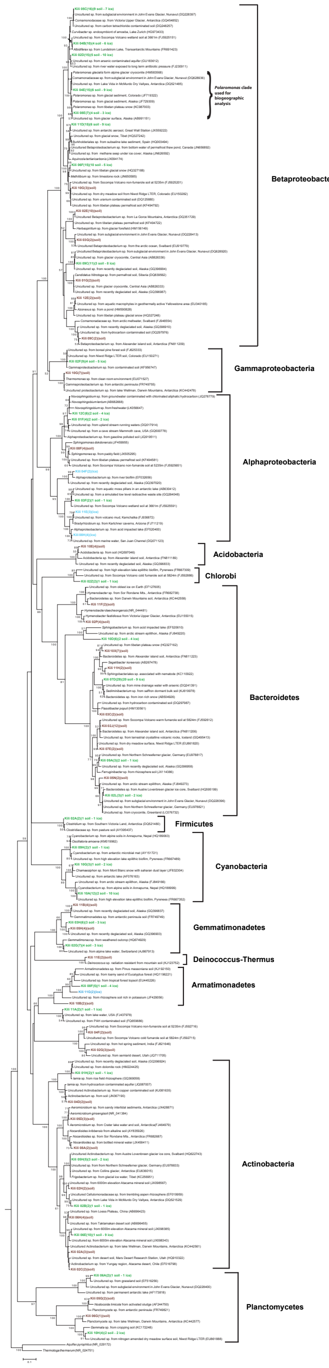

SUPP. FIG. 1. Phylogenetic analysis of 16S rRNA long-read gene sequences retrieved in ice and soil samples close to Mt. Kilimanjaro summit. Maximum Likelihood consensus phylogenetic tree includes 16S rRNA gene sequences retrieved from ice and soil samples close to the summit of Mt. Kilimanjaro and their closest GenBank BLAST and ARB matches. Only OTUs containing > 2 sequences are included. The accession numbers of most closely related taxa are listed parenthetically. Tree is rooted with the sequences of *Aquifex pyrophilus* (NR_029172) and *Thermatoga thermarum* (NR_024751). Kilimanjaro phylotypes are bolded and followed by the number of sequences in each phylotype. Kilimanjaro phylotypes color code is as follows: blue, sequences retrieved in ice; brown, sequences retrieved in soil; green, sequences retrieved in both ice and soil. Node support is given as maximum likelihood values (n. of bootstrap replicates) when equal or greater than 50%. The scale bar corresponds to 0.1 substitutions per site. Major groups are shown to the right. The parenthesis indicates the *Polaromonas* clade that was used for the biogeographic analysis.

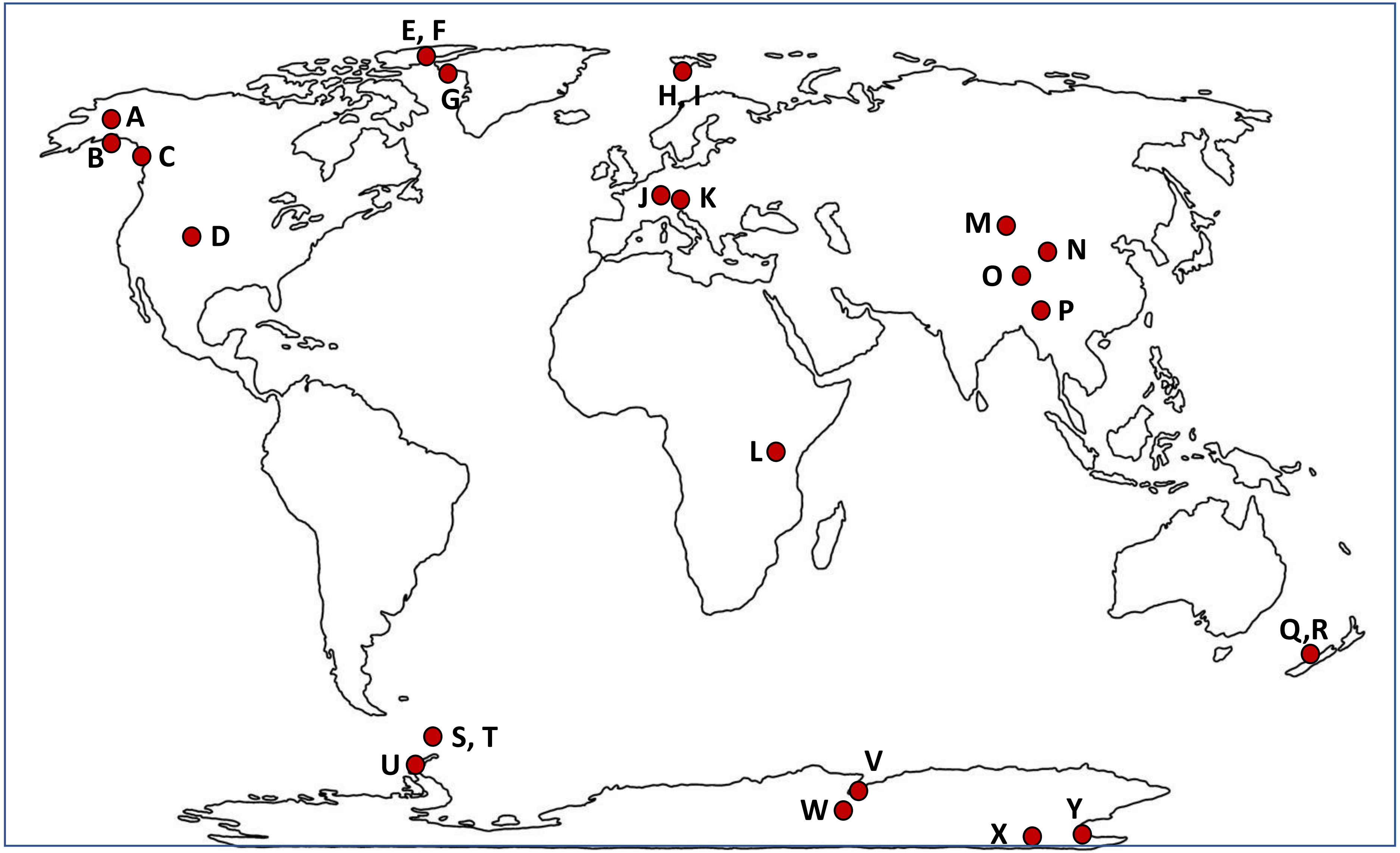

SUPP. FIG. 2. Locations of worldwide sampling sites of *Polaromonas* sequences. Alphabetic labels (A – Y) for location markers correspond to those of Supp. Table 2.

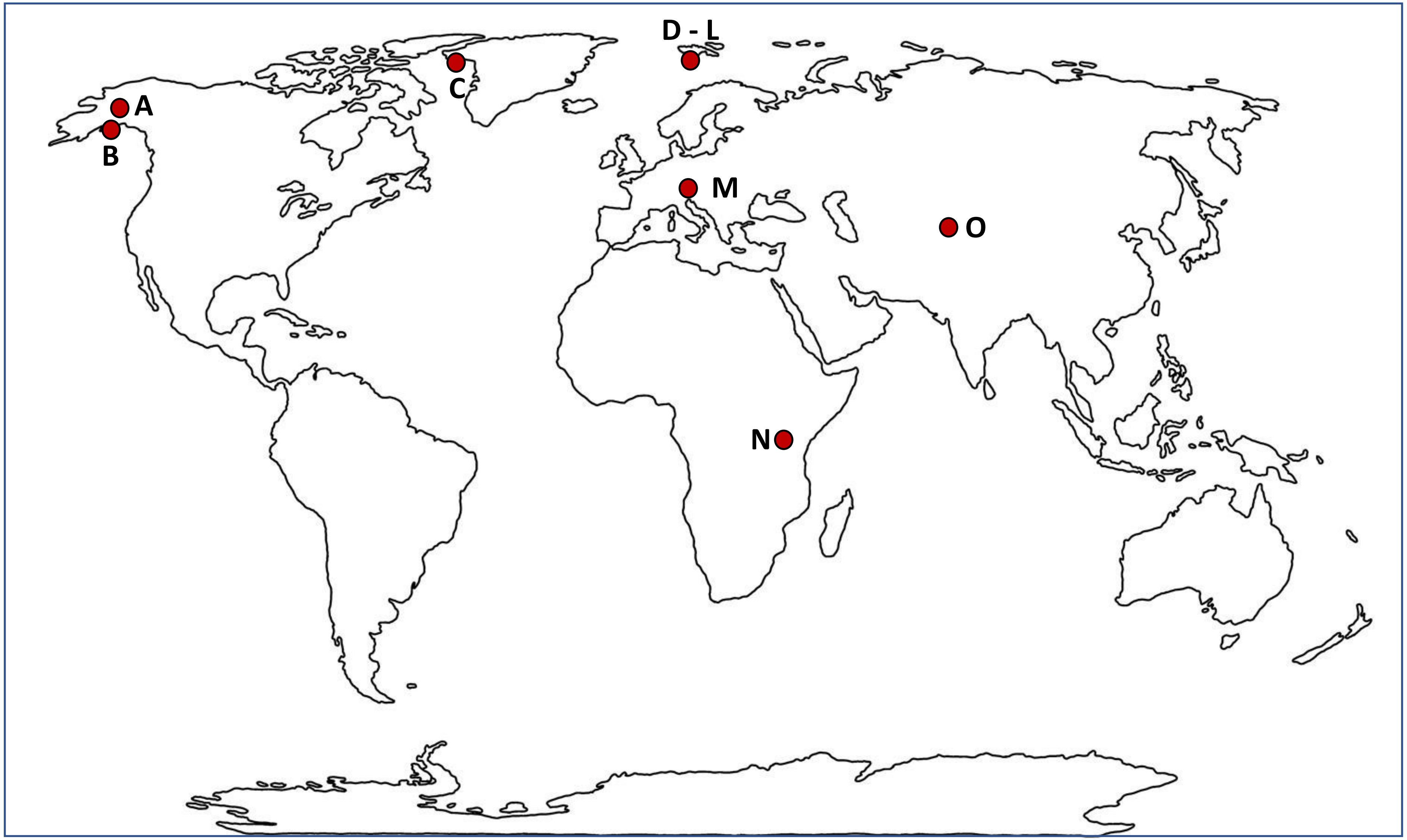

SUPP. FIG. 3. Locations of worldwide sampling sites of *Chlamydomonas* sequences. Alphabetic labels (A – O) for location markers correspond to those of Supp. Table 3.

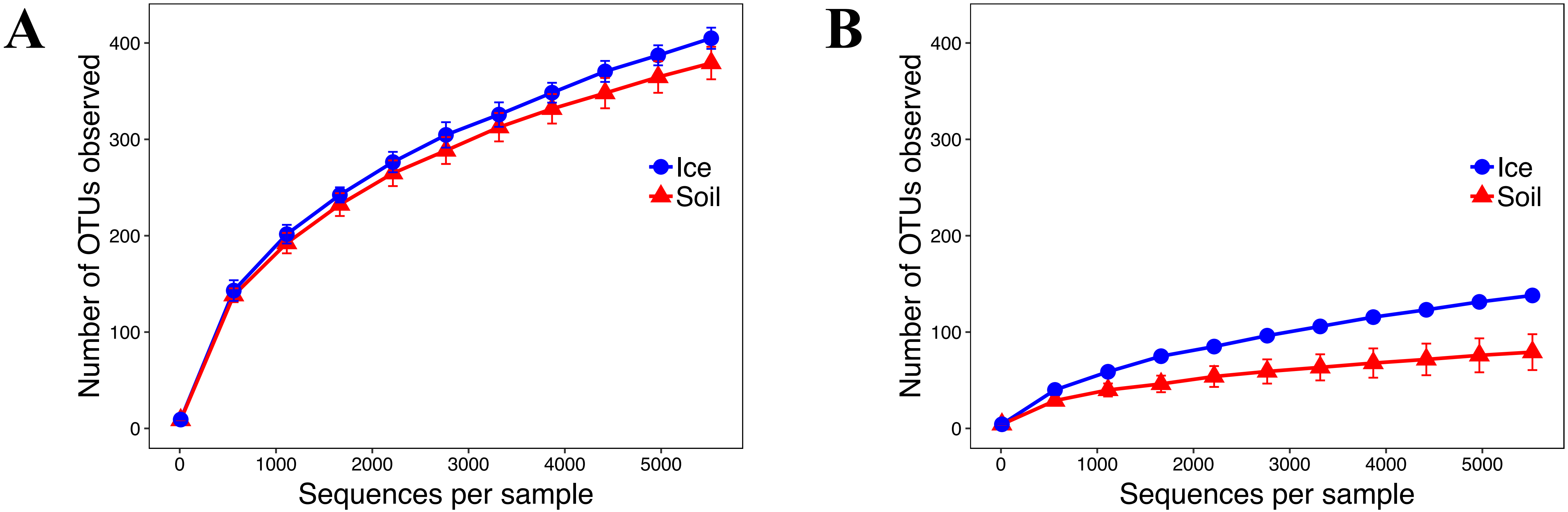

SUPP. FIG. 4. Alpha rarefaction curves based on the number of OTUs observed of 16S (A) and 18S (B) rRNA gene sequences obtained from ice and soil samples close to the summit of Mt. Kilimanjaro. Data shown are means ± s.e. The number of bacterial OTUs observed is comparable in ice and soil samples and close to saturation at the sequencing depth used. On the other hand, fewer eukaryotic OTUs are present in soil samples compared to ice. The metric reveals that sampling saturation is reached at a smaller sequencing depth for soil samples compared to ice for eukaryotes.

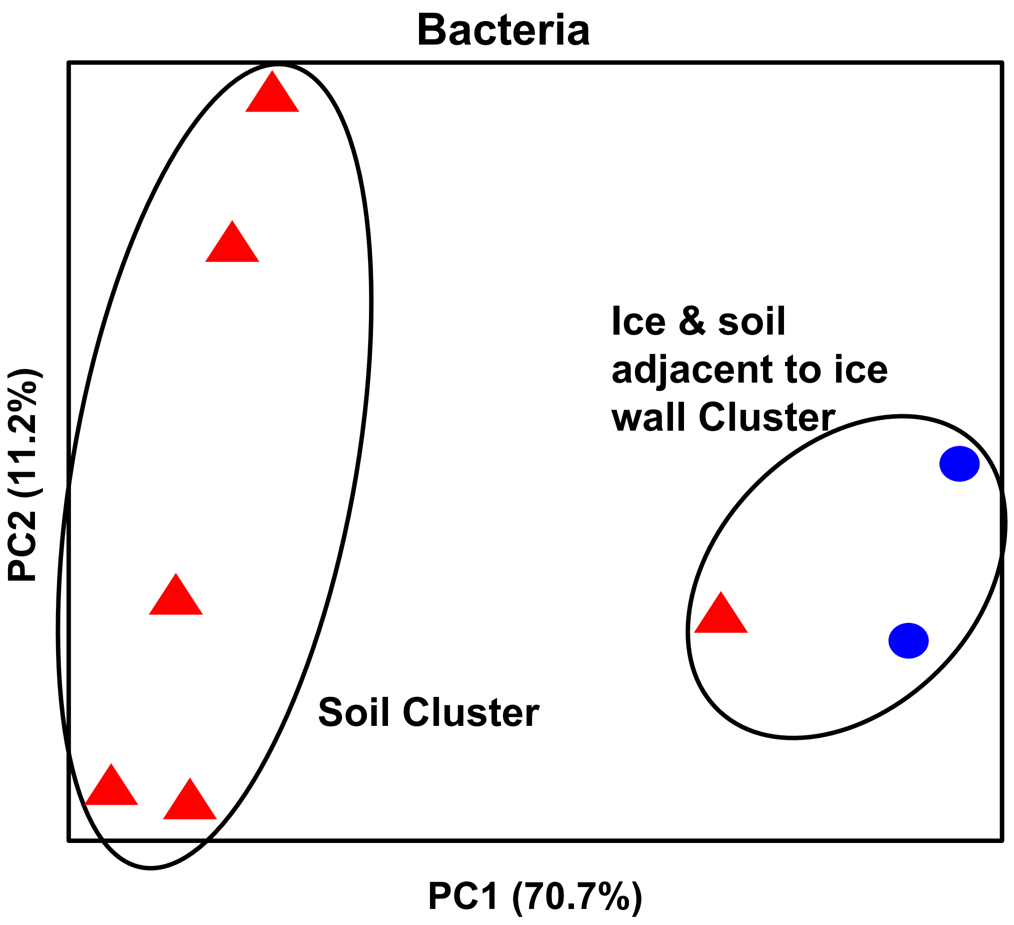

SUPP. FIG. 5. Cluster diagram-based PCoA (Principal Coordinate Analysis) using weighted Unifrac of Bacteria of ice and soil samples retrieved close to Mt. Kilimanjaro summit. The variance explained in each axis is given in parenthesis. Colors indicate samples from ice (blue) and soil (red). Bacterial ice communities are significantly different from soil communities (ANOSIM R = 0.8, P = 0.03). Bacterial communities from the same habitat are more similar to each other than they are to the other habitat type with the exception of the soil sample closest to the ice wall that clusters with the ice samples.

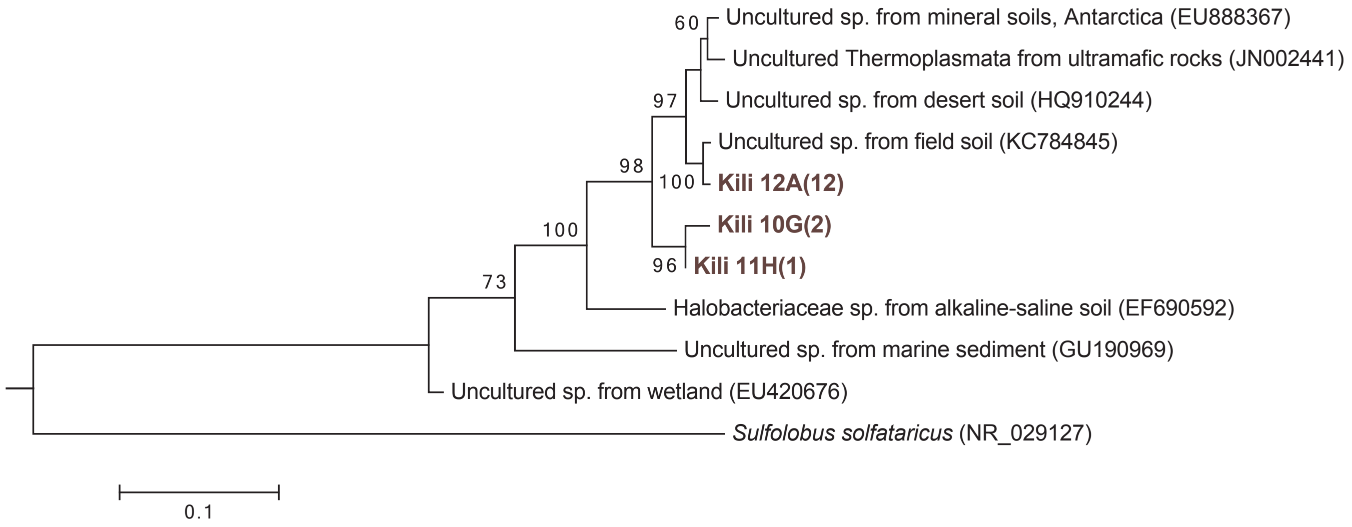

SUPP. FIG. 6. Phylogenetic analysis of 16S rRNA gene archaeal sequences retrieved in a soil sample (N7) close to Mt. Kilimanjaro summit. Maximum likelihood consensus phylogenetic tree includes 16S rRNA gene archaeal sequences from soil close to the summit of Mt. Kilimanjaro and their closest GenBank BLAST matches. The accession number of most closely related taxa are listed parenthetically. Tree is rooted with the sequence of *Sulfolobus solfataricus* (NR_029127). Kilimanjaro phylotypes are bolded in brown and followed by the number of sequences in each phylotype of the library. Node support is given as maximum likelihood values (n. of bootstrap replicates) when equal or greater that 50%. The scale bar corresponds to 0.1 substitutions per site.

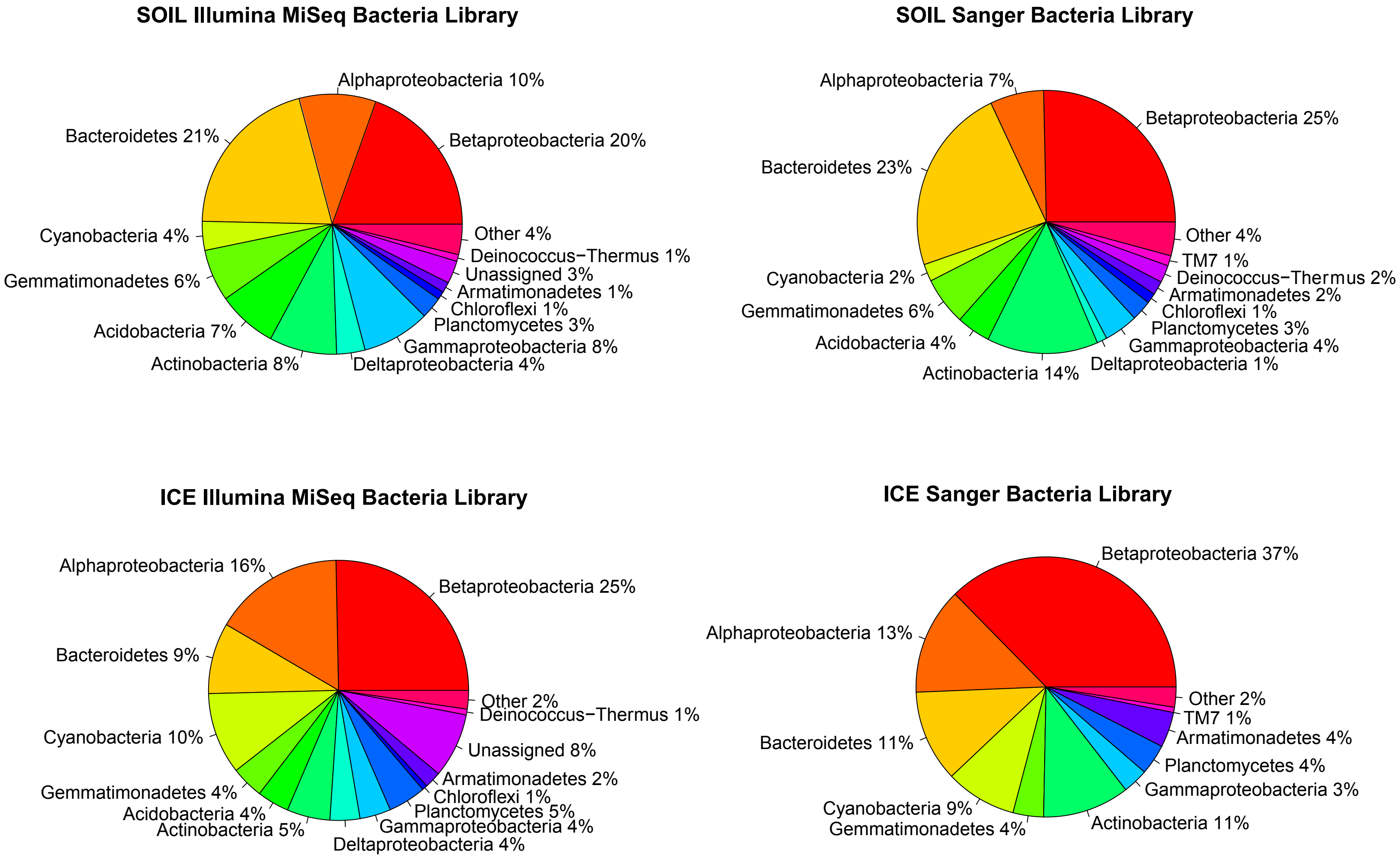

SUPP. FIG. 7. Comparison between broad-level phylogenetic affiliation of Kilimanjaro ice (samples N3 and N8) and soil (samples N1, N5 and N7) bacterial sequences obtained with Illumina MiSeq and Sanger sequencing technologies.

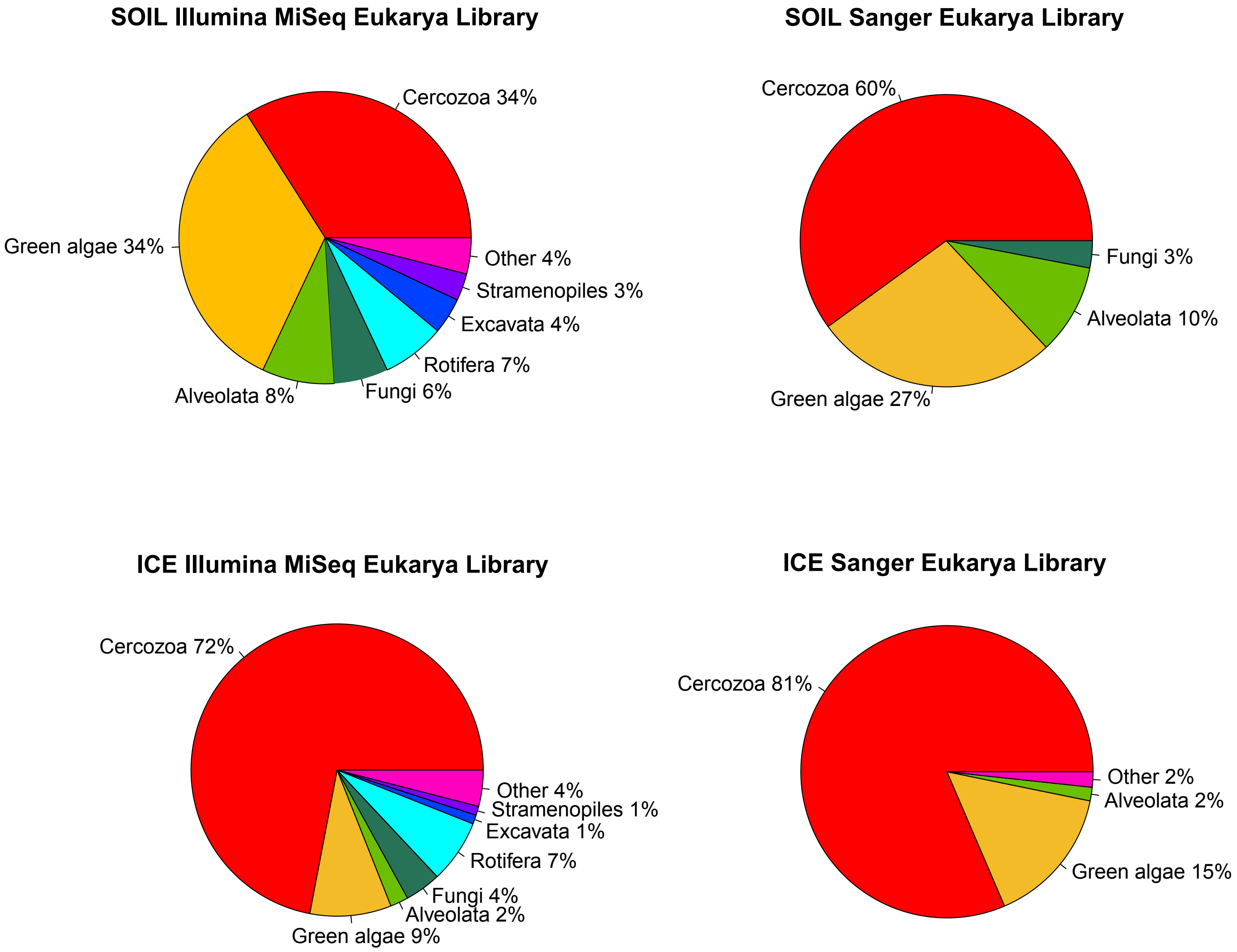

SUPP. FIG. 8. Comparison between broad-level phylogenetic affiliation of Kilimanjaro ice (samples N3 and N8) and soil (samples N1, N5 and N7) Eukaryotic sequences obtained with Illumina MiSeq and Sanger sequencing technologies. Illumina Miseq (pie charts on left) show more phyla than Sanger libraries (pie charts on the right). Eukaryotic libraries are heavily dominated by Cercozoa in both habitats, in particular in the ice.

SUPP. TABLE 1. Environmental classification of long-read bacterial OTUs from ice and soil samples close to the summit of Mt. Kilimanjaro.

**ENDEMIC**						**NON-ENDEMIC**			
**Polythermal OTUs**	Total n. = 46	**Non-Cryophilic OTUs**	Total n. = 11	**Cryophilic OTUs**	Total n. = 3	**Subnovel OTUs**	Total n. = 3	**Novel OTUs**	Total n. = 3
OTU	Phylum	OTU	Phylum	OTU	Phylum	OTU	Phylum	OTU	Phylum
Kili05C(16)	Betaproteobacteria	Kili12C(6)	Alphaproteobacteria	Kili10X(7)	Bacteroidetes	Kili10D(6)	Bacteroidetes	Kili02Z(2)	Chlorobi
Kili04B(10)	Betaproteobacteria	Kili04F(2)	Alphaproteobacteria	Kili05N(2)	Bacteroidetes	Kili02G(3)	Unclassified	Kili08F(5)	Armatimonadetes
Kili02D(15)	Betaproteobacteria	Kili11E(3)	Alphaproteobacteria	Kili02B(2)	Actinobacteria	Kili02H(2)	Actinobacteria	Kili11G(2)	Armatimonadetes
Kili04E(15)	Betaproteobacteria	Kili08H(4)	Alphaproteobacteria						
Kili08E(7)	Betaproteobacteria	Kili11H(2)	Bacteroidetes						
Kili11D(15)	Betaproteobacteria	Kili11E(2)	Deinococcus						
Kili06F(15)	Betaproteobacteria	Kili11A(2)	Unclassified						
Kili10G(3)	Betaproteobacteria	Kili04D(2)	Actinobacteria						
Kili02E(10)	Betaproteobacteria	Kili06A(2)	Planctomycetes						
Kili03G(2)	Betaproteobacteria	Kili05G(2)	Planctomycetes						
Kili09C(11)	Betaproteobacteria	Kili10H(4)	Planctomycetes						
Kili01G(2)	Betaproteobacteria								
Kili12E(2)	Betaproteobacteria								
Kili09C(2)	Betaproteobacteria								
Kili02F(9)	Gammaproteobacteria								
Kili10G(7)	Gammaproteobacteria								
Kili01F(4)	Alphaproteobacteria								
Kili08F(4)	Alphaproteobacteria								
Kili03F(2)	Alphaproteobacteria								
Kili10E(4)	Acidobacteria								
Kili11F(2)	Bacteroidetes								
Kili02P(4)	Bacteroidetes								
Kili07(29)	Bacteroidetes								
Kili03C(2)	Bacteroidetes								
Kili02J(12)	Bacteroidetes								
Kili07E(2)	Bacteroidetes								
Kili09A(3)	Bacteroidetes								
Kili02L(3)	Bacteroidetes								
Kili02A(2)	Firmicutes								
Kili09H(2)	Cyanobacteria								
Kili10G(3)	Cyanobacteria								
Kili10A(12)	Cyanobacteria								
Kili11B(4)	Gemmatimonadetes								
Kili03H(6)	Gemmatimonadetes								
Kili05H(4)	Gemmatimonadetes								
Kili02G(7)	Gemmatimonadetes								
Kili10B(2)	Armatimonadetes								
Kili04F(2)	Unclassified								
Kili01H(2)	Actinobacteria								
Kili05D(3)	Actinobacteria								
Kili08A(2)	Actinobacteria								
Kili05H(5)	Actinobacteria								
Kili06H(4)	Actinobacteria								
Kili06E(10)	Actinobacteria								
Kili02A(3)	Actinobacteria								
Kili02C(2)	Actinobacteria								

SUPP. TABLE 2. Locations, accession numbers, and publication sources for *Polaromonas* sequences.

REGION	SITE	GEOGRAPHIC COORDINATES	ACCESSION N.	PUBLICATION SOURCE	ON SUPP. FIG. 2
Alaska, USA	Toklat Glacier	63.39 N 149.91 W	JF719324-28, 30-38, JF729309	Darcy *et al*., 2011	A
	Byron Glacier	60.74 N 148.85 W	AB991151	Murakami *et al*., 2015	B
	Mendenhall Glacier	58.435837 N 134.5546 W	GQ396863, 949, 971	Sattin *et al*., 2011	C
Colorado, USA	Arikaree Glacier	40.057276 N 105.6432 W	JF719322, 3, 9	Darcy *et al*., 2011	D
Nunavut, Canada	Johns Evans Glacier	79.66 N 74 W	DQ228403, 9	Skidmore *et al*., 2007	E
Nunavut, Canada	Johns Evans Glacier	79.63 N 74.38 W	DQ628932-40, DQ530258	Cheng *et al*., 2007	F
Greenland	Qaanaaq Glacier	77.5033 N 69.1458 W	LC076717	Uetake *et al*., (unpubl.)	G
Svalbard	Hans Glacier	77.04 N 15.39 E	KU586648, KU586652	Gawor *et al*., 2016	H
	Werenskiold Glacier	77.075 N 15.34 E	MG098816	Ciok *et al*. (unpubl.)	I
Germany	Schneeferner Glacier	47.42 N 10.98 E	EU978852	Simon *et al*., 2009	J
Austria	Pitztaler Joechl Glacier	46.55 N 10.53 E	NR_109012-13	Margesin *et al*., 2012	K
Tanzania	Kilimanjaro, 5772 m.a.s.l.	3.04839 S 37.21628 E	KX771285-86, KX771324-26, KX771367, KX771372, KX771376, KX771395, KX771538, KX771602-13	This study	L
Tianshan, China	Glacier n.1	43.15 N 86.87 E	EF423322, 30, 40	Wang *et al.* (unpubl.)	M
	Glacier n.1	43.15 N 86.87 E	FJ979854, 9	Zhang *et al.* (unpubl.)	M
China	Glacier in Gansu Province	39.7 N 96.62 E	JX950030, 31	Liu *et al.* (unpubl.)	N
Tibet, China	Puruogangri Ice Field	33.89 N 89.15 E	DQ227793	Zhang *et al*., 2008	O
	Glacier	29.45 N 96.5 E	JX949585	Liu *et al*. (unpubl.)	P
New Zealand	Franz Joseph Glacier	43.48 S 170.21 E	AY315174, 5, 8	Foght *et al.,* 2004	Q
	Fox Glacier	45.51 S 170.14 E	AY315176, 7	Foght *et al*., 2004	R
Antarctica	Carlini station, King George Island	62.14 S 58.4 W	KY190582, KY190658, KY190735, KY190746, KY190773, KY190785	Vazquez *et al.* (unpubl.)	S
	Baranowski Glacier, King George Island	62.12 S 58.27 W	MG098808	Ciok *et al.* (unpubl.)	T
	Palmer Station, Anvers Island	64.46 S 64.02 W	5 sequences, unpubl.	Vimercati *et al.* (unpubl.)	U
	Davis Station	68.34 S 77.58 E	JX196642	Xiong *et al.* (unpubl.)	V
	Collins Glacier	73.21 S 66.97 E	EU636025-27, EU636029	Garcia-Echauri *et al.*, 2011	W
	Kamb Ice Stream	82.25 S 145 E	FJ477327	Lanoil *et al*., 2009	X
	Lake Vida, Dry Valleys	77.23 S 161.56 E	DQ521547	Mosier *et al.*, 2007	Y

SUPP. TABLE 3. Locations, accession numbers, and publication sources for *Chlamydomonas* sequences.

REGION	SITE	GEOGRAPHIC COORDINATES	ACCESSION N.	PUBLICATION SOURCE	ON SUPP. FIG. 3
Alaska	Toklat Glacier	63.39 N 149.91 W	KM870616-7, KM870646, KM870652, KM870656, KM870662, KM870665-6, KM870675, KM870675, KM870679, KM870685, KM870691, KM870694, KM870731, KM870743-4, KM870752, KM870766, KM870769, KM870771	Darcy et al., 2011	A
	Harding Icefield	60.0031 N 150.007 W	AB902998	Ito et al. (unpubl.)	B
Greenland	Qaanaq Glacier	77.8973 N 64.877 W	AB902971	Ito et al. (unpubl.)	C
Svalbard		77.66 N 14.82 E	JQ790560	Remias et al. (unpubl.)	D
		78.16 N 17.89 E	JQ790557	Remias et al. (unpubl.)	E
		78.18 N 15.48 E	JQ790558	Remias et al. (unpubl.)	F
		79.63 N 10.97 E	AF514411	Leya et al. (unpubl.)	G
		79.27 N 11.77 E	AF514412	Leya et al. (unpubl.)	H
		79.81 N 11.83 E	GU117586	Remias et al., 2010	I
		77.57 N 16.9 E	JQ790559	Remias et al. (unpubl.)	J
		78.97 N 11.58 E	AF514407	Leya et al. (unpubl.)	K
		79.74 N 10.83 E	GU117588	Remias et al., 2010	L
Austria	Tyrol	46.92 N 10.93 E	GU117577	Remias et al., 2010	M
Tanzania	Kilimanjaro, 5772 m.a.s.l.	3.04 S 37.21 E	KX771779-800, KX772005	This study	N
Tajikistan	Pamir	38.7851 N, 72.272 E	AB902973	Ito et al. (unpubl.)	O
